# Simplified Models for the Material Characterization of Cold-Formed RHS

**DOI:** 10.3390/ma10091043

**Published:** 2017-09-06

**Authors:** Carlos López-Colina, Miguel A. Serrano, Miguel Lozano, Fernando L. Gayarre, Jesús Suárez

**Affiliations:** Department of Construction and Manufacturing Engineering, University of Oviedo, Building 7, Campus of Gijón/Xixón, 33203 Asturias, Spain; serrano@uniovi.es (M.A.S.); lozanomiguel@uniovi.es (M.L.); gayarre@uniovi.es (F.L.G.); suarezg@uniovi.es (J.S.)

**Keywords:** FEM, hollow sections, material characterization, cold-formed, stub-column

## Abstract

It is well known that the cold-forming process used to manufacture tubes causes an increase in both the yielding stress and the ultimate strength of the corner material in rectangular steel hollow sections. This may have a significant effect on the resistance of any structure built with those profiles. However, the mentioned material hardening can be difficult to take into account in the calculations for member design or to evaluate the connection resistance through the design formulation or when using numerical simulation models. As an attempt to face the above-mentioned problem, the present paper presents a comparison among simplified approaches that consider homogeneous material properties for the whole section. It has been carried out by comparing the results obtained from the finite element modelling of stub column tests in which the material properties based on the flat faces were considered for the whole profile.

## 1. Introduction

Tubular structures are becoming more and more popular as an alternative to open sections, rather than just for steel structures designed as a lattice girder [1]. The hollow sections that are used for those structural purposes are fabricated either by a hot finishing process or through a cold-forming process. Both types of manufacturing processes result in slightly different characteristics, but practitioners do not have any special preference since they are more concerned by the availability of each type of profile in their region. Nevertheless, the cold-forming process increases the yielding stress and the ultimate strength of the corner material in the rectangular steel hollow sections (RHS). This may affect the resistance of the structures that use these profiles [2,3]. The problem arises when a practitioner wants to take into account the mentioned material hardening in the calculations, since it could be considered for the design of members or to assess the connection strength and stiffness through the design equations or by finite element models. First, the design equations cannot be properly applied to sections with non-homogeneous characteristics. Second, the consideration of different material properties for the corners of the tubes in the finite element simulations has traditionally been limited due to the difficulty of obtaining and testing coupons from those parts of the hollow section. Some research has been conducted in order to address the problem. Some authors focused on the investigation of the corner parts of cold-formed steel sections at elevated temperatures, like Chen et al. [4,5]. Gardner, Zhang, and others [6,7] established the main topic as the comparison of material properties for hot-rolled and cold formed rectangular hollow sections. In the work carried out by Rossi, Afshan, and Gardner [8,9], a study into the prediction of strength enhancements in cold-formed structural sections was presented after an experimental program. The static properties depending on the manufacturing method were compared by Sun and Packer [10]. Nevertheless, most of the previous research was focused on assessing approaches that give a weighted yield limit for the whole section based on the properties of the base material instead of taking the properties of the faces of the finished rectangular hollow sections. This traditional point of view aims the formulation at RHS manufacturers instead of structural practitioners. Equations for considering an averaged value for the material enhancement due to cold-forming that are aimed at structural engineers should be preferably based on the material properties of the faces because the main material property for the design of structures is the nominal yield limit, checked by manufacturers by testing coupons from the faces of the finished hollow sections.

As an attempt to face the above-mentioned problem, the present paper shows a comparison among simplified approaches that consider homogeneous material properties for the whole section based on the face properties. It was carried out by comparing the results obtained from the finite element modelling of stub column tests in which the material properties based on the flat faces were taken for the whole profile.

The finite element model of the stub column tests was initially validated with experimental results from the literature by taking the different material properties for the corners and for the flat faces obtained from tensile tests. Then, the simplified approaches for homogeneous material properties based on the flat faces were compared with both the previously validated FEM model and the experimental tests. These models are discussed in order to choose the one with the best agreement. As an additional support to the model validation, some more experimental results from extra stub column tests carried out by the authors are compared with the FE analysis as well. It is expected that the conclusions pointed out in this research will lead to savings on time and money in the unavoidable task of the material characterization for FE models and strength calculations involving rectangular hollow sections.

## 2. Materials and Methods

### 2.1. Experimental Tests

Stub column tests and tensile tests from two different sources were used in order to compare the validity of different proposals for the simulation of the material properties of rectangular hollow sections. The first source is a previous comprehensive work performed by some Australian researchers for other purposes, and the second one is a set of stub column tests that intends to confirm the results and conclusions of our work.

A set of 13 stub column tests carried out by Wilkinson et al. [11] was used as an experimental support for the validation of the finite element model. This report, created by its authors for different purposes, was suitable for its use in the present paper because they also presented the results of standard tensile tests of coupons taken from faces and corners of the rectangular hollow sections. The force-displacement curves of the stub-column tests and the yield and ultimate strength (*f_y_*, *f_u_*) and strains (*ε_y_*, *ε_u_*) from the tensile tests were used in the present work.

Table 1 shows a summary of the different profiles that were tested by Wilkinson et al. with their geometrical properties. All the specimens tested in this set followed de common rule for stub-column tests that states a length of three times the profile depth.

The results of force-displacement curves and strength of these 13 stub-column tests are compared with the different simplified models for the material properties of cold-formed RHS in the third section.

Five more stub-column tests were carried out in order to confirm the results of the study with our own experimental tests. The profiles tested were chosen among plastic and compact sections of S275 steel since the previously mentioned 13 tests were performed mainly in slender sections and some semi-compact profiles. Four different RHS were tested, and one of the tests was reproduced two times in order to confirm the repeatability of the testing method. Table 2 shows the nominal and measured dimensions of the tested profiles in this test set.

Only tensile coupons from the faces were tested for this set of stub-columns since this experimental work was mainly planned to check the simplified approaches for the material properties of the whole section based on the material properties of the faces. A uniaxial testing machine, model CMED-AR (Sistemas de Ensayo, Madrid, Spain) with a maximum compression load capacity of 2500 kN, was used. The test consists of applying a progressive centered compression load on the short column between both ending faces until the specimen reaches the maximum load and the failure occurs. The specimens were cut to a nominal length of 300 mm. After that, the ending faces were machined to obtain stub columns with perfect flat opposite end surfaces perpendicular to the length of the tube.

The results of the strength for the 18 stub-column tests are presented and compared with the different models in the third and fourth section.

### 2.2. Finite Element Models

**Figure 1:**
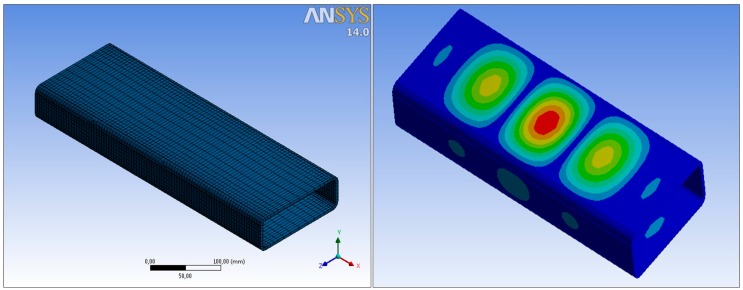
Examples of mesh and first buckling mode used in the FEM.

Four different FE models of the stub column tests were created with Ansys (Workbench V. 14, ANSYS Inc., Canonsburg, PA, USA) for this work. All of them have the following common characteristics:

The Ansys Shell 181 element type was used (Figure 1). This is a typical four-noded quadratic shell element for non-linear problems. This type of element has been found as a suitable element for the simulation of members, connections, and structures made with structural hollow sections. Many examples can be found in previous works [12,13,14].

All the simulations were displacement-controlled by forcing the displacement of one of the ends of the stub columns and fixing the other end [15]. The applied load is obtained from the reactions in the supports.

Geometrical and material non-linearities were considered. It was included as an initial imperfection based on the shape of the first linear buckling mode (Figure 1), being the maximum imperfection the 80% of the manufacturing tolerance. This value was taken from the European standard “Cold formed welded structural sections of non-alloy and fine grain steels” EN 10219 [16].

The material was considered as bi-linear and the plastic tangent modulus (*E_p_*) was calculated from the experimental yield and ultimate strengths and stresses (*f_y_*, *f_u_*, *ε_y_*, *ε_u_* respectively) as in Equation (1).

(1) $$E_{p}=\frac{f_{u}-f_{y}}{\varepsilon_{u}-\varepsilon_{y}}$$

The differences of the four models came from the consideration of the material hardening in the corners.

#### 2.2.1. Model 1: Non-Homogeneous Material

This model tries to simulate the actual behavior of the stub-columns by considering different material properties in the corners and in the faces according to the values presented in Table 2. Only the tests carried out by Wilkinson et al. [11] could be modelled in this way because there were no material properties of the corners for the second set of tests. Just the rounded part of the profiles was considered as a “corner” in the FE model.

#### 2.2.2. Model 2: Eurocode-Based Homogeneous Approach

This FEM uses the Eurocode proposal [17] for the average material properties of cold-formed open profiles. The code gives an average yield limit *f_ya_* from the yield and ultimate strength of the base material (*f_yb_*, *f_ub_*). However, the EN-10219 [16] standard just consider to test the material properties on coupons taken from the finished cold-formed RHS (after the manufacturing process). This circumstance lead us to take the material properties of the faces (*f_yf_*, *f_uf_*) in the Eurocode equations instead of the base material properties. The other parameters that appear in Equation (2) are the thickness (*t*) and the cross section area (*A*), as follows:

(2) $$f_{ya}=f_{yf}+\left(28\times \frac{t^{2}}{A}\right)\times \left(f_{uf}-f_{yf}\right)$$

#### 2.2.3. Model 3: Material Properties of the Faces

This model took the material properties obtained from the faces for all the section, i.e., ignoring any influence of the higher strength in the corners.

#### 2.2.4. Model 4: AISI-Based Homogeneous Approach

**Figure 2:**
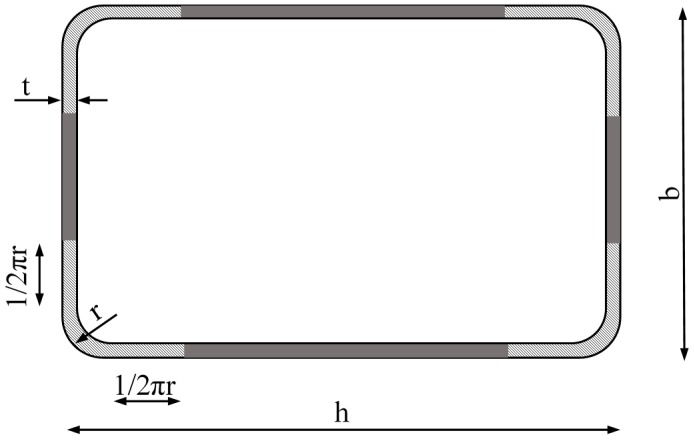
Corner area according to Model 4.

The AISI specification for the material properties of cold formed sections [18], widely proposed by many authors, like Ghersi et al. [19], presents a different equation that is essentially the same proposed by Abdel-Rahman et al. [20]. According to the arguments presented for model 2, the base material properties have been substituted by the material properties of the faces (*f_yf_*, *f_uf_*) in this formulation. The corner zone was considered as the rounded part plus two flat bands 1/2πr wide (see Figure 2). The yield limit in those corner areas can be calculated as shown in Equation (3):

(3) $$f_{yc}=0.4f_{yf}+0.6\frac{B_{c}f_{yf}}{{\left(r/t\right)}^{m}}$$

where *B_c_* and m are two parameters that depend on the *f_ub_*/*f_yb_* ratio, obtained through Equations (4) and (5), respectively.

(4) $$B_{c}=3.69\frac{f_{uf}}{f_{yf}}-0.819{\left(\frac{f_{uf}}{f_{yf}}\right)}^{2}$$

(5) $$m=0.192\frac{f_{uf}}{f_{yf}}-0.068$$

The calculated value of yield stress in the corner *f_yc_* is then used to obtain a weighted average value for the whole section according to the lengths stated in Figure 2 and Equation (6).

(6) $$f_{ya}=\frac{6\pi r}{2h+2b-8r+2\pi r}f_{yc}+\frac{2h+2b-8r-4\pi r}{2h+2b-8r+2\pi r}f_{yf}$$

## 3. Results

The results of standard tensile tests on coupons cut from flat faces and corners are shown in Table 3. This third table presents the results of yield limit and ultimate strength of the adjacent faces to the welded face (Adj1 and Adj2) and the opposite face to the weld (Opp). The coupons from corners has been referred as C1, C2, C3, and C4.

The results of yield and ultimate stress (*f_y_*, *f_u_*) of standard tensile tests on coupons cut from flat faces are shown in Table 4. One flat coupon from the front face (the minor face) and one flat coupon from one lateral face were tested. Results are identical for O01 and O05 because they are material properties obtained from the same coupons since both stub-column tests used the same 150 × 100 × 6 RHS profile.

### Comparison of Results

**Figure 3:**
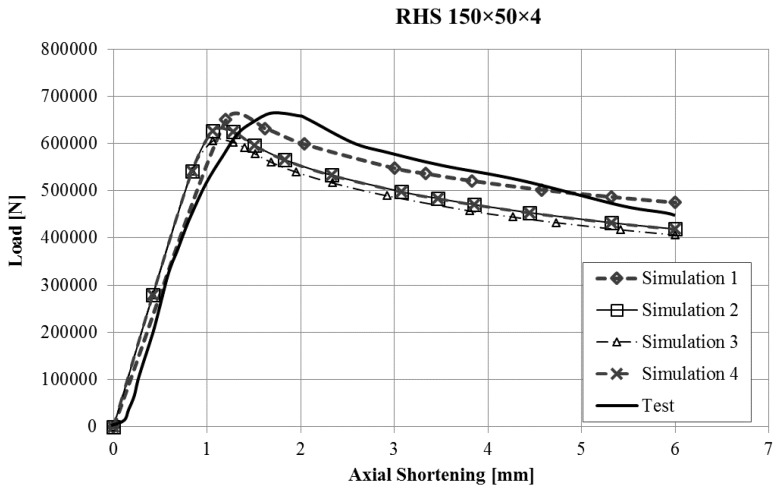
Force-displacement curves of specimen S02.

**Figure 4:**
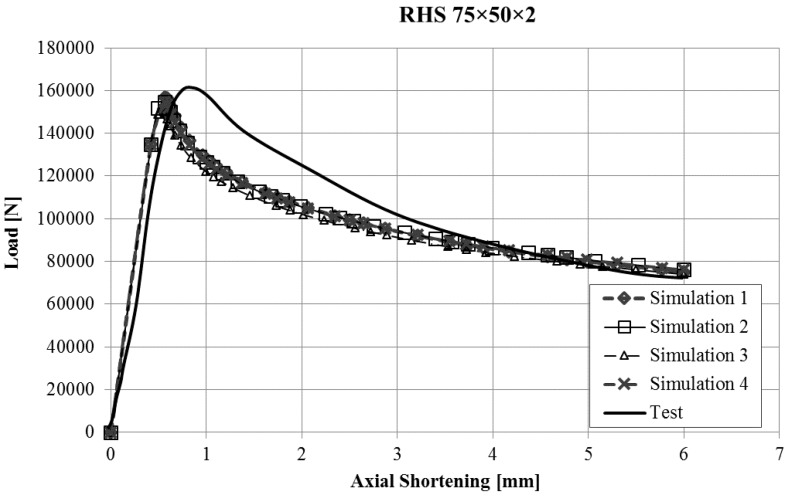
Force-displacement curves of specimen S07.

**Figure 5:**
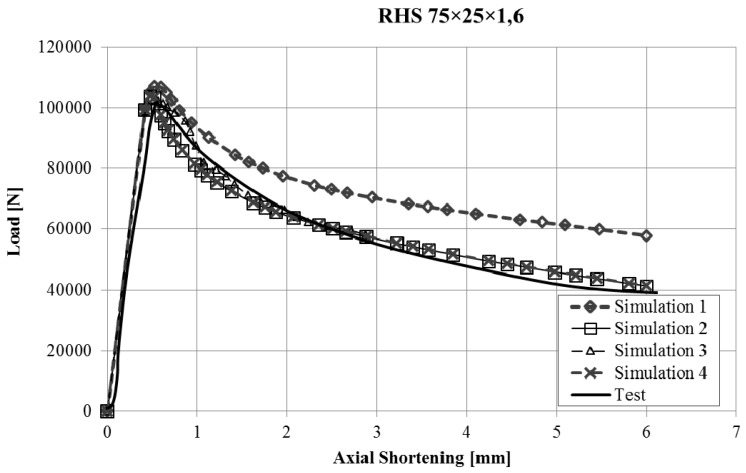
Force-displacement curves of specimen S10.

**Figure 6:**
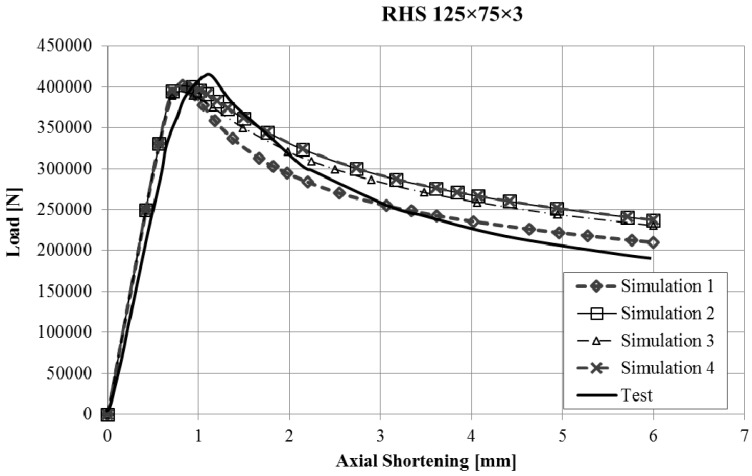
Force-displacement curves of specimen S13.

The force-displacement curves obtained with the finite element model of the stub-column tests were compared with the experimental results. Reasonably good agreement was obtained for all the tests, and in some cases, the results were very good, taking into account the inherent uncertainties that arise in this kind of simulations (geometrical imperfections, thickness changes, residual stresses, etc.). Since it would not be operative to present the curves for all the studied cases in this article, Figure 3, Figure 4, Figure 5 and Figure 6 present the force displacement curves of four stub-column cases (S02, S07, S10, S13) showing some representative shapes. The corresponding results of the four FE simulations with the different material models are drawn in the same graph in order to assess them.

The real testing conditions cause initial displacements almost without load in some tests (see Figure 3). If we take this into account, our simulations reproduce the shape of experimental force-displacement curves quite well, especially before the maximum load is reached. It is worth noting that Model 2 and Model 4 give curves that almost overlap in all the studied cases.

**Figure 7:**
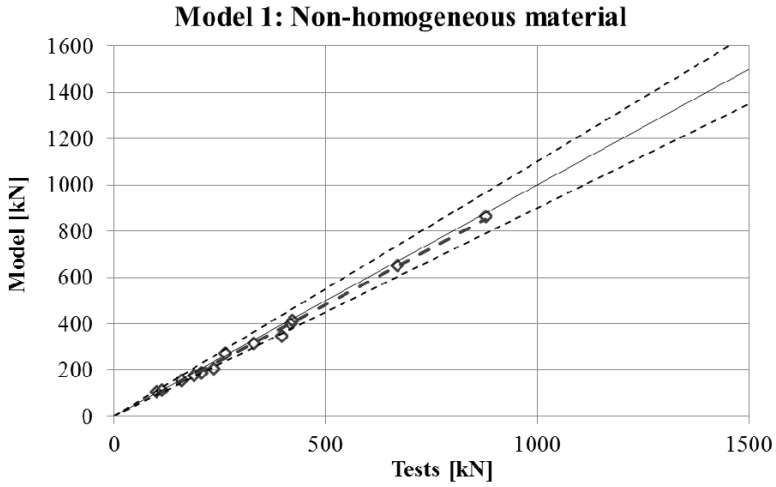
Comparison between strengths from tests and FEM with material model.

**Figure 8:**
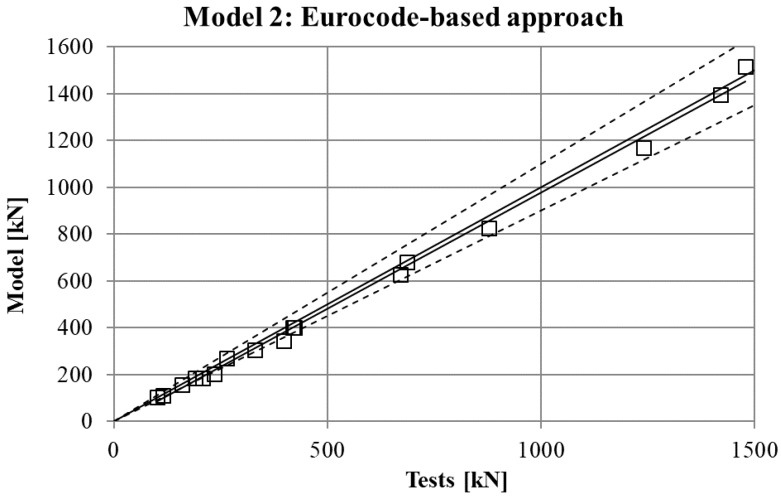
Comparison between strengths from tests and FEM with Model 2.

**Figure 9:**
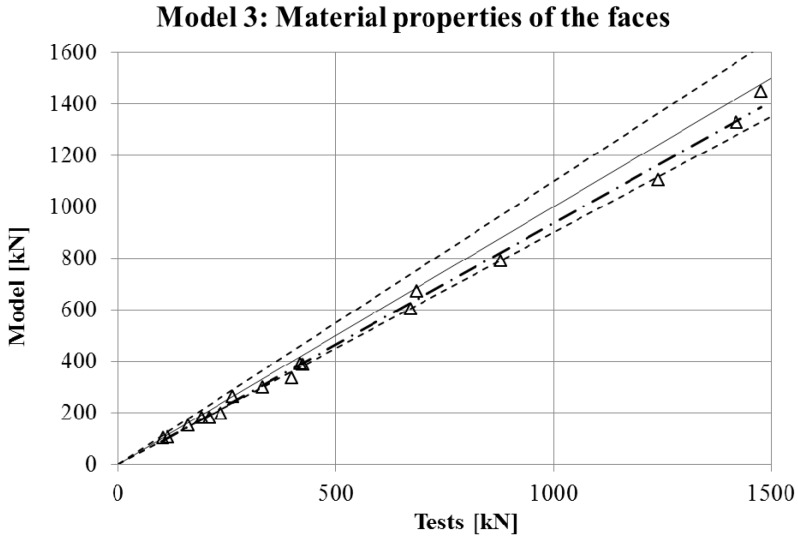
Comparison between strengths from tests and FEM with Model 3.

**Figure 10:**
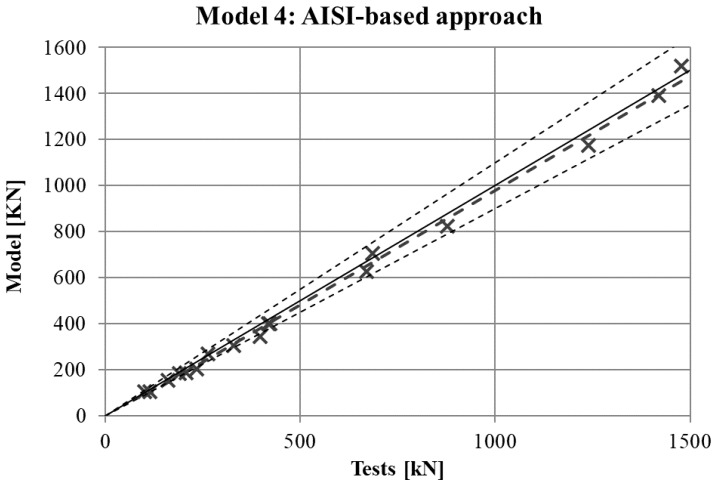
Comparison between strengths from tests and FEM with Model 4.

The maximum force in the force-displacement curve gives the strength of the stub-column. The values of strength obtained in experimental tests are compared in Figure 7, Figure 8, Figure 9 and Figure 10 for the four FE simulations with different material models. A simple linear regression was plotted for each set of comparison points in order to visually evaluate the difference between the grey line that represents a perfect match and the set of results. Additionally, two dotted lines represent a separation of 10% from the ideal result.

## 4. Discussion

Two conclusions can be obtained from the Figure 3, Figure 4, Figure 5, Figure 6, Figure 7, Figure 8, Figure 9 and Figure 10. First, the graphs show that the four proposed models applied to the previously presented FE simulation give slightly conservative results. Second, it is clear that the worst model for the material properties is the one that does not take into account the material properties of the corners and only accounts for the properties of the flat faces for the whole section (Figure 9). However, there are no clear differences among the other three figures (Figure 7, Figure 8, and Figure 10) so there is no clear preference among them.

In order to study which one is actually the best approach, the results of strength obtained from the experimental tests and the four FE models are presented in Table 5. As was expected, the best results in all cases were obtained from Model 1 (non-homogeneous material considering the actual properties of corners), with average deviations of about 5%. This can be considered as accurate enough to validate Model 1 as a reference simulation. In addition, the simplest model (Model 3), which does not consider the influence of the hardening of the corners, gives the worse results in all cases, as was expected. However, the three simplified approaches (Models 2–4) gave similar results to Model 1.

The last column of the table presents the best approach between Model 2 and Model 4, showing that both proposals give almost equal results in most of cases, and a clear preference cannot be determined.

## 5. Conclusions

Based on the results of this study, the following conclusions can be drawn.

The proposed finite element model with shell elements, bi-linear material properties, and the consideration of non-linear buckling with an initial imperfection based on the first linear buckling mode is accurate enough to obtain good results in the non-linear simulation of RHS until local buckling or yielding failure.

The main standards for the technical delivery requirements of cold formed hollow sections [16] only consider tensile tests on coupons taken from the faces of the finished RHS to obtain the material properties of RHS. In addition, this is the basis for the nominal yield strength that is certified by the manufacturers, this nominal value being the only datum that a practitioner usually manages to calculate or simulate the structural behavior. However, existing equations for the strength enhancement in cold-formed RHS are designed to calculate a weighted yield limit by considering the material properties of the base material before the cold-forming process [17], and the all previous research work [2,3,4,5,6,7,8,9,10] assessed these equations following this rule. Since this is a point of view that is useful for manufacturers but not for practitioners, the present article has studied some simplified rules (models 2, 3, and 4) to calculate an average yield stress that consider the material properties of the faces of the finished RHS, leading to conclusions that are useful for common practice of structural calculations and current finite element simulations.

Four models for the consideration of the material properties were tested in the finite element simulations. Model 1 considers the different material properties in the corners according to real values from tensile tests carried out with coupons from that part of the tubes. As it was expected, this simulation obtained the best results when it was compared with the experimental tests. However, no big differences were found between Model 1 and models 2 and 4, which use adaptations of the equations proposed in the Eurocode and AISI standards to consider an averaged yield limit for the whole section. Model 3 is the traditional simple simulation that considers just the material properties of the flat faces for the whole section.

The equations proposed in models 2 and 4 use the material properties of the faces of the RHS instead of the material properties of the base material proposed in the standards. In fact, since it is expected that the material of the faces would have slightly lower values of *f_y_* and *f_u_* before the cold-forming, the use of the face characteristics after cold-forming would be preferable. Another practical reason for this is the fact that standards like EN-10219 [16] only mention the material characterization through tensile tests for the finished cold-formed profiles and not for the base material.

Models 2 and 4 behave similarly and they can both be used for the material properties of the RHS sections to obtain better results in FE modelling without testing the corners. However, the conservative approach that considers only the material properties of the faces is precise enough for practitioners and designers of structures.

## Figures and Tables

**Table 1:** Dimensions of specimens of the tests by Wilkinson et al. [11].

Specimen	Depth *h* (mm)	Width *b* (mm)	Thickness *t* (mm)	Radius *r* (mm)
**S01**	150	50	5	7.5
**S02**	150	50	4	6
**S03**	150	50	3	4.5
**S04**	150	50	2.5	3.75
**S05**	150	50	2.3	3.45
**S06**	100	50	2	3
**S07**	75	50	2	3
**S08**	75	25	2	3
**S09**	75	25	1.6	2.4
**S10**	75	25	1.6	2.4
**S11**	150	50	3	4.5
**S12**	100	50	2	3
**S13**	125	75	3	4.5

**Table 2:** Measured dimensions of specimens.

Specimen	Nominal Dimensions	Depth *h* (mm)	Width *b* (mm)	Thickness of Faces *t* (mm)	Thickness of Corners *t*_c_ (mm)
**O01**	150 × 100 × 6	150.0	100.0	5.72	6.40
**O02**	150 × 100 × 4	150.5	99.8	3.75	4.22
**O03**	200 × 150 × 6	200.0	151.0	5.70	5.98
**O04**	100 × 100 × 6	100.0	100.0	5.93	6.37
**O05**	150 × 100 × 6	150.0	100.0	5.71	6.08

**Table 3:** Material properties of the tests by Wilkinson et al. [4].

Specimen	Nominal Material	*f_y_*; *f_u_* Adj1 (MPa)	*f_y_*; *f_u_* Adj2 (MPa)	*f_y_*; *f_u_* Opp (MPa)	*f_y_*; *f_u_* C1 (MPa)	*f_y_*; *f_u_* C2 (MPa)	*f_y_*; *f_u_* C3 (MPa)	*f_y_*; *f_u_* C4 (MPa)
**S01**	C450	425; 492	457; 498	505; 543	520; 556	480; 566	495; 566	500; 560
**S02**	C450	460; 537	454; 516	514; 580	563; 616	545; 601	550; 608	570; 613
**S03**	C450	445; 520	442; 506	514; 585	-	540; 588	545; 594	540; 611
**S04**	C450	445; 527	446; 518	485; 560	540; 600	530; 585	545; 596	540; 588
**S05**	C450	453; 528	434; 508	480; 547	535; 585	470; 523	490; 526	480; 530
**S06**	C450	445; 492	452; 505	480; 540	490; 538	490; 540	510; 560	500; 547
**S07**	C450	345; 430	411; 484	428; 482	475; 520	450; 500	445; 480	465; 503
**S08**	C450	438; 507	475; 522	487; 537	480; 538	550; 588	530; 576	525; 585
**S09**	C450	450; 506	428; 515	487; 545	560; 589	555; 576	555; 605	565; 585
**S10**	C350	421; 460	423; 452	445; 514	505; 540	500; 515	510; 532	510; 555
**S11**	C350	370; 433	369; 425	380; 436	465; 511	475; 516	480; 534	495; 542
**S12**	C350	395; 454	404; 445	433; 471	500; 536	485; 530	480; 520	505; 545
**S13**	C350	396; 450	397; 448	403; 453	470; 510	475; 518	475; 508	470; 520

**Table 4:** Material properties of the second set of tests.

Specimen	Nominal Material	*f_y_* Front (MPa)	*f_u_* Front (MPa)	*f_y_* Lat (MPa)	*f_u_* Lat (MPa)	*f_y_* Avg (MPa)	*f_u_* Avg (MPa)
**O01**	S275	479	536	456	530	468	534
**O02**	S275	389	468	369	456	379	462
**O03**	S275	400	475	394	471	397	473
**O04**	S275	473	536	496	549	485	543
**O05**	S275	479	536	456	530	468	534

**Table 5:** Comparison of results of strength.

Specimen	Strength (kN)					Best Approach between Models 2 and 4
	Tests	Model 1	Model 2	Model 3	Model 4	
S01	878	864	826	792	822	2
S02	670	651	626	605	626	2 = 4
S03	422	414	400	388	400	2 = 4
S04	330	314	306	299	307	4
S05	263	273	269	263	269	2 = 4
S06	235	205	203	199	203	2 = 4
S07	190	187	183	180	184	4
S08	160	157	154	151	155	4
S09	114	113	109	106	106	2
S10	101	107	103	102	104	4
S11	397	348	345	336	346	4
S12	207	189	185	181	186	4
S13	418	402	400	390	401	4
O01	1420	-	1394	1329	1393	2
O02	685	-	681	672	705	2
O03	1477	-	1513	1451	1477	4
O04	1239	-	1168	1107	1239	4
O05	1408	-	1394	1329	1393	2
